# Every Body Likes Her

**Published:** 1891-05

**Authors:** 


### EVERY BODY LIKES HER.

There is a type of girl that every body likes. Nobody can tell exactly why, but after you have met her you turn away to some other woman and say: "Don't you like Miss Grosvenor?" Now, the reason you like her is a subtle one; without knowing all about her you feel just the sort of a girl she is.

She is the girl who is not "too bright and good" to be able to find joy and pleasure all over the world.

She is the girl who appreciates the fact that she cannot always have the first choice of every thing in the world.

She is the girl who is not aggressive and does not find joy in inciting aggressive people.

She is the girl who has tact enough not to say the very thing that will cause the skeleton in her friend's closet to rattle his bones.

She is the girl who, whether it is warm or cold, clear or stormy, finds no fault with the weather.

She is the girl who, when you invite her to any place, compliments you by looking her best.

She is the girl who is sweet and womanly to look at and listen to, and who doesn't strike you as a poor imitation of a demimondaine.

She is the girl who makes this world a pleasant place because she is so pleasant herself.

And, by and by, when you come to think of it, isn't she the girl who makes you feel she likes you, and therefore, you like her?—*Boston Globe.*

